# Comparison between the standard and low-dose chest CT scans on the lung quantitative analysis in critically ill patients

**DOI:** 10.1186/cc12052

**Published:** 2013-03-19

**Authors:** S Froio, D Chiumello, I Cigada, M Brioni, S Coppola, F Menga, L Gattinoni

**Affiliations:** 1 Fondazione IRCCS Ca' Granda - Ospedale Maggiore Policlinico, Milan, Italy; 2 Università degli Studi di Milano, Milan, Italy

## Introduction

Quantitative analysis of a lung CT scan is considered the reference method to study lung recruitability to optimize the ventilatory strategy in ARDS patients. However, CT implies a risk of radiation exposure, especially when CT analysis is necessary to monitor the evolution of ARDS. The aim of this study was to evaluate the impact of a lower radiation dose on lung CT quantitative analysis compared with analysis computed by standard CT.

## Methods

Sedated and paralyzed patients underwent two chest CT scans: a Standard CT (120 kV, 110 mAs, pitch 1.2, collimation 0.6 mm; Care Dose Technology) and a CT performed by a lower radiation dose (Lowdose: 120 kV, 60 mAs). CTs were performed during the same inspiratory or expiratory hold, for different values of airway pressure. Each CT image was manually delineated excluding pleural effusions and mediastinal structures. We analyzed Standard and Lowdose CT images with dedicated software to quantify the tissue weight of lung regions with different degrees of inflation. Lung quantitative data computed by Standard and by Lowdose scans were compared according to Bland-Altmann analysis.

## Results

**Figure 1:**
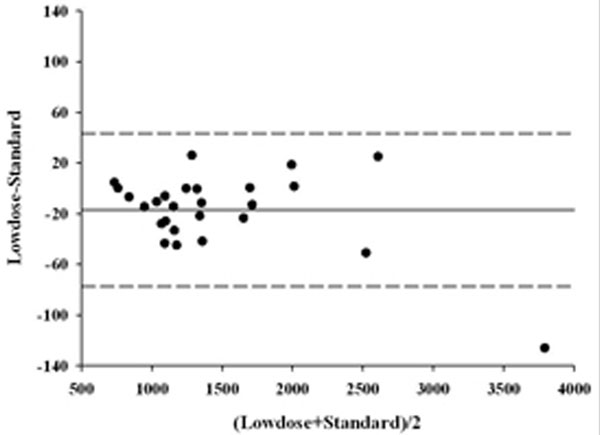
**Total tissue weight**.

**Figure 2:**
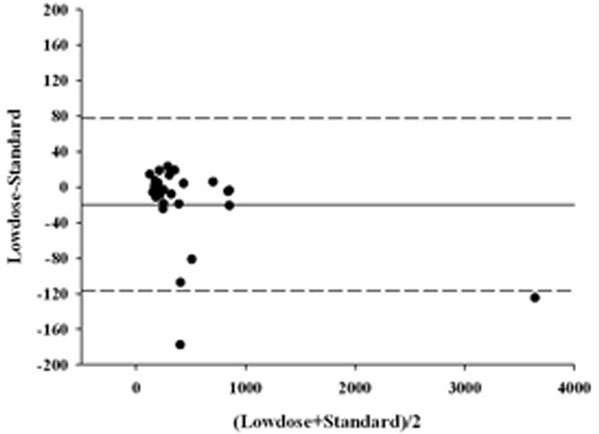
**Not inflated tissue weight**.

We enrolled 13 patients admitted to our ICU. In the Bland-Altman analysis of the lung total tissue and not inflated tissue, the bias and agreement bands for Standard CT scan and Lowdose CT quantitative analysis were -16.97 g (-77.43 to +43.49 g) and -9.65 g (-116.81 to 77.51 g), respectively (Figures 1 and 2).

## Conclusion

Lung CT quantitative analysis computed by Lowdose CT scans could be a useful tool for monitoring and ventilatory management of ARDS patients.
